# Evaluation of an nhs juvenile idiopathic arthritis (JIA) treatment pathway compared to published international recommendations

**DOI:** 10.1186/1546-0096-12-S1-P178

**Published:** 2014-09-17

**Authors:** Katherone L Green, Marinka Twilt, Taunton Southwood

**Affiliations:** 1 Paediatric Rheumatology, Birmingham Children's Hospital, UK; 2 Paediatric Rheumatology, NHS, Birmingham, UK

## Introduction

The ACR recommendations for the treatment of Juvenile Idiopathic Arthritis (ACR-JIA) were published in 2011 with the aim of providing an evidence-based therapeutic pathway for effective JIA treatment.

## Objectives

Our aim was to determine the feasibility of applying ACR-JIA to a real-life paediatric JIA cohort and to evaluate the treatment pathway of those children.

## Methods

We conducted a retrospective analysis of a single-centre paediatric JIA cohort. This included a review of the patient case notes, radiology and drug monitoring data of all children with JIA diagnosed with multi-joint (5 or more joint) disease in the 2 years since ACR-JIA were published.In total, 35 patients fulfilled ILAR criteria for the diagnosis of JIA since 2011: systemic arthritis (n=5), polyarthritis (n=25) and extended Oligoarthritis (n=5). Duration to Methotrexate and Etanercept treatments was calculated, and the frequency of drug monitoring noted.

## Results

25 females and 10 males (median age at onset 13, range 1.5-15 years) were included in the evaluation. Median age at disease onset for poly/extended oligoarthritis was 10 years (1.5-16), with a median of 12 joints (12-38) active at presentation, and for the systemic group median age at onset was 6 years (2-7), with a median number of 6 active joints (2-10). 3 polyarthritis patients were rheumatoid factor positive (0 extended oligoarthritis and 0 systemics). 22/30 patients with polyarthritis/extended oligoarthritis followed the ACR recommendations for treatment according to their disease severity, commencing methotrexate therapy within a median of 6 weeks (3-37) of diagnosis and etanercept (where relevant) within a median of 7 months (3-24) of diagnosis. 7 patients did not follow ACR-JIA guidelines due to experiencing excessive durations (for disease severity) between diagnosis and commencing methotrexate or etanercept treatment. One patient did not have sufficient regular drug monitoring tests. All patients with systemic arthritis followed the ACR-JIA recommendations for treatment and drug monitoring.

## Conclusion

Overall, 27/35 patients followed the ACR-JIA recommendations. This evaluation highlights the difficulty of achieving rapid commencement of new JIA therapies and the challenging of regular drug monitoring.

## Disclosure of interest

None declared.

**Table 1:** 

JIA subtype	Disease severity	N=	Poor prognostic factors	RhF positive	Median duration to MTX	Median duration to etanercept
***Systemic***	Low	1	0	0	N/A	N/A
		
N=5	Moderate	2	0	0		
		
	High	2	0	0		

						

***Polyarthritis & extended oligoarthritis***	Low	0	0	0	6 (3-37)	7 (3-24)
		
N=30	Moderate	23	9	2		
		
	High	6	2	1		
		
	Unknown	1	0	0		

